# Physical and Chemical Characterization and Bioavailability Evaluation In Vivo of Amaranth Protein Concentrate

**DOI:** 10.3390/foods12081728

**Published:** 2023-04-21

**Authors:** Yuliya S. Sidorova, Nikita A. Petrov, Irina B. Perova, Alexey I. Kolobanov, Sergey N. Zorin

**Affiliations:** Federal Research Centre of Nutrition and Biotechnology, 109240 Moscow, Russia; petrov-nikita-y@mail.ru (N.A.P.); erin.feather@yandex.ru (I.B.P.); alleexxkl@yandex.ru (A.I.K.); zorin@ion.ru (S.N.Z.)

**Keywords:** amaranth, pseudocereals, protein, amino acid score, true digestibility, functional food, biological value, squalene

## Abstract

Special attention is being paid to the study of amaranth proteins. They are characterized by a high biological value that significantly exceeds those of grain crops. The production of protein concentrate from amaranth flour includes preliminary enzymatic hydrolysis, extraction of the resulting mixture, protein precipitation, microfiltration, and freeze-drying. In our study, the obtained amaranth protein concentrate was limited by valine, with an amino acid score of 74%. The true digestibility of the amaranth protein concentrate determined in vivo was 97.6 ± 0.3%, which was significantly lower than that of casein (99.3 ± 0.2%). The protein digestibility-corrected amino acid score value of the concentrate was 72.2%. The obtained concentrate was a rich source of selenium, copper, magnesium, manganese, and iron. Ferulic acid was the only polyphenolic compound found in the amaranth protein concentrate, but its content was significantly greater compared to the original flour. Saponins were not removed completely during the process of obtaining the amaranth protein concentrate. We identified 15 saponins in the concentrate, mainly of the bidesmoside type, the sapogenins of which are related derivatives of oleanolic acid. Thus, the developed amaranth protein concentrate can be used as an ingredient in functional food products, with a high biological value.

## 1. Introduction

Nutrition, which is balanced by the composition of macro- and micronutrients, as well as minor biologically active substances that are adequate for physiological needs, is the most important factor determining the state of health of the human body. Thus, the development of approaches to the complex processing of raw agricultural materials and the creation of new functional foods of high nutritional and biological value is relevant. New functional food products must fully meet the requirements of safety and efficacy, which require scientific substantiation of the composition and a complex preclinical evaluation. At present, technologies are constantly being improved, and the range of plant-based health food products is expanding, including food products with amaranth flour and amaranth grain components (protein, starch, oil, and minor biologically active substances) in the composition.

Amaranth, like quinoa or buckwheat, is one of the most widely used pseudocereals in nutrition and is now cultivated in many countries. The genus *Amaranthus* L. is represented by more than 70 species [1]. There are approximately 900 botanical units of this plant, including subspecies and varieties (both wild and cultivated) [2]. The high nutritional and biological value of amaranth grain, even called the culture of the 21st century, determines the main directions of scientific research. These directions include the following:

- First, to the identification and quantitative assessment of the macro- and micronutrients and minor biologically active food substances (so-called phytonutrients—secondary metabolites of plant origin) in its composition;
- Second, the development of technological approaches to grain processing, extraction, and concentration of biologically active compounds;
- Third, the development of technology and creation of food products mainly based on flour or concentrates/isolates of amaranth grain protein.

Special attention is being paid to the characterization of amaranth proteins. Almost all publications emphasize their high biological value, as determined by the balance of the amino acid composition, which significantly exceeds that of grain crops [3,4,5]. However, analyzing the data presented in the works of various research centers reveals that the amino acid score (determined by the ratio of essential amino acids and their specific content) varies in a fairly wide range, from 80% to 100% [6,7]. Basically, valine, leucine, and isoleucine appear as limiting amino acids for amaranth grain protein. According to other estimates, however, amaranth protein is limited in aromatic and sulfur-containing amino acids.

The biological value of protein isolates or concentrates obtained from amaranth grain is characterized in the vast majority of recent studies on the basis of amino acid composition analysis and does not include balance studies in vivo, which seems to be incorrect. Obviously, for a correct quantitative assessment of amaranth protein’s biological value, its true digestibility (determined in vivo in metabolic experiments) should be taken into account based on the protein digestibility-corrected amino acid score (PDCAAS), which is equal to the amino acid score multiplied by the true digestibility [8]. An important feature of amaranth protein is the absence of gluten in its composition, which allows it to be used as a food product for people suffering from celiac disease.

The lipid profile of amaranth grain includes saturated, monounsaturated, and polyunsaturated fatty acids, triglycerides, sterols, phospholipids, glycolipids, and tocopherols [9]. According to scientific publications, linoleic acid makes up more than 50% of the total fatty acids in amaranth oil: oleic—more than 25%, palmitic—approximately 20%, and α-linolenic—approximately 1%. Squalene, an intermediate compound in cholesterol biosynthesis, is the most important biologically active substance in amaranth. Squalene is effectively used for the prevention and dietary therapy of lipid metabolism disorders. Amaranth oil is the main plant source of squalene; its content in amaranth oil can vary from 3% to 8%.

Amaranth grain contains a unique set of phytonutrients—compounds that protect plants from aggressive environmental influences—such as saponins, phenolic compounds (including flavonoids), phytosterols, and other compounds. The following main groups of phenolic compounds have been identified in the most cultivated amaranth species (*A. caudatus*, *A. cruentus*, and *A. hypochondriacus*): phenolic acids (ferulic, p-coumaric, and p-hydroxybenzoic), flavonoids (rutin and quercetin), and tannins [10,11,12], both individually and in the composition of extracts [13,14,15,16]. According to [17], 100 g of amaranth grain provides over 50% of the recommended dietary intake of copper, iron, manganese, magnesium, phosphorus, and zinc. Amaranth flour, along with buckwheat and corn flour, are rich sources of selenium.

Thus, the aim of our study was to obtain an amaranth protein concentrate, to determine its amino acid score in vitro, and to conduct an in vivo metabolic experiment on growing laboratory rodents to determine its true digestibility and protein efficiency ratio (PER), as well as to evaluate the fatty acid, mineral substance, phenolic compound, saponin, and squalene content of the resulting concentrate.

## 2. Materials and Methods

### 2.1. Materials

All chemicals and solvents were of analytical grade.

Celloviridin G20X (activity 3000 units/g) and Prozyme 4G (activity 15,000 units/cm^3^) were used. Celloviridin G20X is a complex of carbohydrase enzymes of the *Trichoderma reesei (viride)* strain that is capable of deep destruction of both cell walls and the following individual plant polysaccharides: cellulose, glucan, xylan, hemicellulose, and other non-starch polysaccharides. By destroying the walls of plant cells, the Celloviridin G20x enzyme complex increases the availability of starch, protein, and fat.

Prozyme 4G is a bacterial enzyme preparation standardized for glucoamylase activity. Glucoamylase sequentially hydrolyzes α-1,4- and α-1,6-glycosidic bonds, cleaving off glucose residues from the non-reducing ends of starch molecules, dextrins, and oligosaccharides, which are the end products of hydrolysis.

Commercial amaranth flour was produced by OOO Russkaya Oliva (Saint-Petersburg, Russia). The manufacturer obtains flour from amaranth grain of its own amaranth variety, “Voronezh”. “Voronezh” is one of the earliest maturing varieties among the world’s amaranth collection (vegetation period up to 100 days). It is perfectly cultivated in different climatic conditions due to its resistance to cold. A smaller amount of green mass and low growth help to harvest the plant. The seeds are spherical and white, and the endosperm is mealy.

### 2.2. Chemical Composition of Amaranth Flour and Protein Concentrate

Protein content was determined according to the Kjeldahl method, with premineralization using a conversion factor from total nitrogen to protein equal to 6.25 [18], with an automatic analyzer (Kjeltec 8100; FOSS Analytical AB, Höganäs, Sweden). Sample moisture was determined using a Mettler Toledo MJ33 moisture analyzer (Mettler Toledo, Greifensee, Switzerland). Ash content was determined according to [19]. Squalene content was determined according to [20].

### 2.3. Mineral Composition of Amaranth Flour and Protein Concentrate

The content of 12 elements was measured in amaranth flour and amaranth protein concentrate using inductively coupled plasma mass spectrometry (ICP-MS) with a 7700 series instrument manufactured by Agilent Technologies (Santa Clara, CA, USA). The mineralization of samples was performed with concentrated nitric acid and concentrated hydrogen peroxide at a ratio of 5:1 in an automated microwave sample preparation system (“TOPWAVE”; Analytik Jena, Jena, Germany). Standard solutions and analytical curves were used for each element.

### 2.4. Determination of Lipid Content

Extraction and determination of total lipid content were carried out according to the Folch method [21]. For the determination of fatty acid composition, conditions of gas chromatography with a flame ionization detector were used. Injection volume 1 µL, split mode 50:1, carrier gas hydrogen, and flow rate 1.65 mL/min. Injector temperature 200 °C and detector temperature 240 °C. Separation conditions: Use an initial temperature of 100 °C, isotherm 3 min, increase at a rate of 3 °C/min to 200 °C, isotherm 3 min, increase at a rate of 3 °C/min to 240 °C, then isotherm 6 min. Data collection and processing were carried out using Agilent ChemStation Rev.B.04.03 and Microsoft Excel 2007 software, respectively. The ratio of fatty acids was determined using the internal normalization method.

### 2.5. Determination of Flavonoid Profile

The study was carried out using an Ultimate 3000 liquid chromatography system with a diode array spectrophotometric detector (DMD) and a triple quadrupole mass spectrometric detector (MSD) (TSQ Endura). HPLC-DMD conditions: Phenomenex Luna C18 150 × 4.6 mm column with a particle size of 5 µm; mobile phase A—0.1% solution of formic acid in water, B—0.1% solution of formic acid in acetonitrile; gradient elution: 0–35 min 15–60% B, 35–40 min 60% B, 41–50 min 15% B; eluent flow rate 0.5 mL/min; column temperature 30 °C; autosampler temperature 20 °C; volume of injected sample 5 µL; DMD at λ = 350 nm and λ = 338 nm; spectra were taken in the wavelength range of 200–400 nm. Data processing was carried out using Thermo Xcalibur 4.2.47 software.

### 2.6. Determination of Hydroxycinnamic Acid Profile

HPLC-DMD conditions: Phenomenex Luna C18 150 × 4.6 mm column with a particle size of 5 µm; mobile phase A—0.1% solution of formic acid in water, B—0.1% solution of formic acid in acetonitrile; gradient elution: 0–18 min 10–25% B, 18–30 min 25–40% B, 30–35 min 40–60% B, 41–50 min 10% B; eluent flow rate 0.5 mL/min; column temperature 30 °C; autosampler temperature 20 °C; volume of injected sample 5 µL; DMD at λ = 330 nm and λ = 310 nm; spectra were taken in the wavelength range of 200–400 nm. Data processing was carried out using Thermo Xcalibur 4.2.47 software.

### 2.7. Determination of Triterpene Saponins Profile

The profile of triterpene saponins was determined using reverse-phase HPLC-MS on an Ultimate 3000 liquid chromatography system with a diode array spectrophotometric detector (DMD) and a TSQ Endura triple quadrupole mass spectrometric detector. Stationary phase: Phenomenex Luna C18(2) 250 × 4.6 mm column with a particle size of 5 µm, mobile phase A—0.1% formic acid in water, B—0.1% formic acid in acetonitrile, gradient elution mode: 0–35 min 15–60% B, 35–40 min 60% B, 41–50 min 15% B. The samples were extracted with 60% aqueous methanol in an ultrasonic bath at room temperature for 30 min, then centrifuged, and the supernatant was analyzed. Saponins were identified by retention times and mass spectra in accordance with [20].

### 2.8. Determination of Amino Acid Composition

The amino acid composition was determined using a Hitachi automatic amino acid analyzer, followed by computer data processing using Multichrome software for Windows. Preparation of samples was carried out according to the method of Moore and Stein [20]. The samples dehydrated with acetone and defatted with sulfuric ether were hydrolyzed with a 6 N hydrochloric acid solution for 24 h at a temperature of 105 °C. The hydrolyzates were evaporated to dryness in a water bath, and the dry residue was dissolved in 0.02 N HCl.

For the quantitative determination of tryptophan, the alkaline hydrolysis method was used. This method is based on treating the object with alkali, followed by a color reaction with p-dimethylaminobenzaldehyde, and measuring the developing color on a spectrophotometer at 610 nm.

The calculation of amino acid score is conducted as follows:

(1) $$\mathrm{AS}=\frac{\mathrm{AA}}{{\mathrm{AA}}_{\mathrm{st}}}\times 100$$

where AS is the amino acid score (%), AA is the essential amino acid content (g/100 g of protein), and AA_st_ is the content of the same amino acid (g/100 g of standard protein) according to the FAO/WHO scale.

### 2.9. Preparation of Amaranth Protein Concentrate

**Figure 1:**
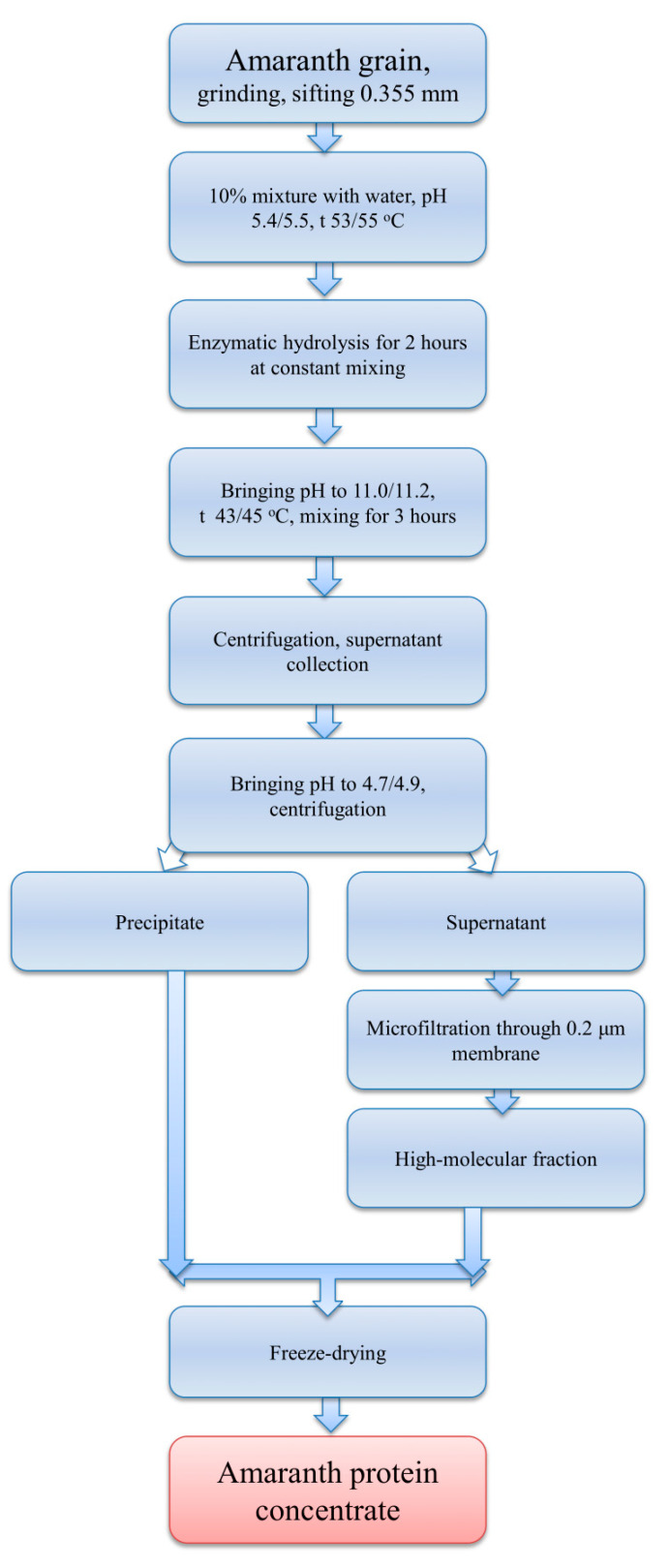
Principal scheme for preparation of amaranth protein concentrate.

The amaranth protein concentrate was obtained according to the technological scheme presented in Figure 1.

We used commercial amaranth flour to avoid the stage of grinding the grain. A mixture of amaranth flour with water (ratio 1 kg of flour/10 L of water) was treated for 2 h (pH 5.4–5.5, T = 54–55 °C) with a mixture of enzyme preparations (1.2 g of Celloviridin G20X and 1.2 mL of Prozyme 4G) in an autoclavable reactor (FA-10, PROINTECH, Putshino, Russia). Upon completion of the enzymatic hydrolysis, the pH of the mixture was adjusted with alkali to 11.0–11.2, and the subsequent 3 h protein extraction was carried out at T = 43–45 °C with constant stirring. The resulting suspension was centrifuged (J-6B BECKMAN centrifuge; Brea, CA, USA, 3500 rpm, 35 min), and the pH of the supernatant was adjusted to 4.7–4.9 with 0.1 M hydrochloric acid to precipitate proteins, followed by centrifugation. The precipitate was separated and additionally washed with water. Microfiltration was used to remove low-molecular compounds using a micro- and ultrafiltration unit based on an ASF-018 filter holder (VLADISART, Vladimir, Russia). The precipitate was combined with the microfiltrate and freeze-dried (freeze-dryer LS-500; PROINTECH, Putshino, Russia).

### 2.10. Animal Study

The experiment was carried out on 24 growing male Wistar rats with an initial average body weight of 80 ± 5 g. All animal procedures were conducted according to standard requirements [22]. Animal studies were approved by the Ethics Committee of the Federal Research Centre of Nutrition and Biotechnology (protocol code No. 11, 15 December 2021) in accordance with the standard principles described in “Guide for the Care and Use of Laboratory Animals”. The animals were adapted in the laboratory for 7 days before the start of the experiment. During this period, daily examinations of the external conditions of the animals were carried out. Animals without signs of health deviations were included in the experiment. The animals were kept individually in polycarbonate cages with 12/12 h dark/light cycle and T = 25 ± 1 °C. The animals were divided into 2 groups: a control group (No. 1) (*n* = 12 rats) with a body weight of 117 ± 2 g and an experimental group (No. 2) (*n* = 12 rats) with a body weight of 118 ± 3 g (*p* ≥ 0.05). The animals in both groups received a basic isocaloric (376 ± 3 kcal/100 g of dry food) and isonitrogen (20 ± 2% protein by calorie content) semisynthetic diet [23]. The animals in the control group (No. 1) received a diet in which casein was used as the protein source. The animals in the experimental group (No. 2) received the same semisynthetic diet, but casein was completely replaced by amaranth protein concentrate.

The animals received water and food ad libitum. During the 15 days of the study, food intake and body weight gain of each animal were determined; food intake was monitored every day, and animals were weighed 2 times a week. From days 14 to 15, during the so-called “metabolic” period, the amount of nitrogen in the food and that excreted in feces were determined.

The body weight gain and the PER were determined for each rat. The body weight gain of the animals was determined in grams per 1 g of protein consumed, and PER was determined using Formula (2):

(2) $$\mathrm{PER}=\frac{W_{t}-W_{0}}{I_{p}}=\frac{\Delta W}{I_{p}}$$

where Δ*W* is the weight gain of the rat (in grams) during the experimental period, *W_t_* is the body weight of the rat (in grams) on the last day of the experimental period, *W*_0_ is the body weight of the rat (in grams) on the first day of the experimental period, and *I_p_* is the amount of protein consumed by the rat (in grams) during the experimental period.

### 2.11. Comparative Determination of the True Digestibility of Casein and Amaranth Protein Concentrate

The method for calculating the true nitrogen digestibility (Dtr) is based on determining the proportion of nitrogen truly absorbed in the gastrointestinal tract of the rat (Atr), expressed as a percentage of the nitrogen consumed by the animal with food (I). The value of the true digestibility of nitrogen corresponds to the value of the true digestibility of the protein. The amount of nitrogen excreted in feces during the day by a rat on a protein-free diet was taken equal to 0.023 g [24].

The true protein digestibility was calculated using Formula (3):

(3) $$D_{tr}=\frac{I-\left(F-F_{k}\right)}{I}\times 100\%=\frac{A_{tr}}{I}\times 100\%$$

where *D_tr_* is the true digestibility (%), *I* is the total amount of nitrogen consumed by the rat with food during the “balance” period (g), *F* is the amount of nitrogen excreted with the feces during the “balance” period (g), *F_k_* is the amount of nitrogen excreted in feces by a rat that was on a protein-free diet during the same “balance” period (g), and *A_tr_* is the true amount of nitrogen absorbed in the gastrointestinal tract of a rat during the “balance” period (g).

PDCAAS is equal to the amino acid score of the first limited amino acid multiplied by the true digestibility.

(4) $$\mathrm{PDCAAS}=\frac{{\mathrm{AS}}_{1}\times D_{\mathrm{tr}}}{100}$$

where AS_1_ is the amino acid score of the first limited amino acid (%), and D_tr_ is the true digestibility (%).

The true protein digestibility and PDCAAS were calculated individually for each rat.

### 2.12. Statistical Analysis

Statistical analysis of the obtained data was performed using SPSS Statistics version 20.0 software (IBM, Armonk, New Yourk, USA) and Microsoft Excel for Windows. Mean (M), standard deviation (SD), and standard error of the mean (m) were calculated. The probability of accepting the null hypothesis about the coincidence of the distributions of the compared samples was determined using the two-tailed Student’s *t*-test for pairwise-related values and the Mann-Whitney nonparametric post hoc test. Data are presented as M ± m. The critical significance level of the null hypothesis (*p*) was taken equal to 0.05.

## 3. Results

### 3.1. Amaranth Flour and Amaranth Protein Concentrate Characterization

Table 1 shows the chemical composition of the original amaranth flour and the resulting amaranth protein concentrate.

The protein content was increased by 2.5 times compared to the raw material.

The squalene content in the flour was 19.9 ± 4.9 mg/100 g. The squalene content in the obtained concentrate was 21.4 ± 3.0 mg/100 g.

The results of determining the amino acid composition of the amaranth protein concentrate, as well as the assessment of its amino acid score relative to the ideal protein scale [3], are presented in Table 2.

The calculation of the amino acid score showed that amaranth protein concentrate is limited by valine, which has a score of 74%.

The amount of micro- and macroelements contained in amaranth protein concentrate is important from the point of view of its possible use in the composition of food products (Table 3).

From the data presented in Table 3, it follows that the content and redistribution of micro- and macroelements in amaranth protein changed during the process of concentrate production. As a result of microfiltration, the content of calcium, potassium, phosphorus, magnesium, manganese, iron, and zinc decreased in the concentrate, while the content of copper, chromium, cobalt, and selenium increased. The accumulation of sodium was probably due to the addition of 1 M NaOH during the pH adjustment step of protein leaching.

Table 4 shows the profile of fatty acids in the original amaranth flour and in amaranth protein concentrate.

The technology used to obtain amaranth protein concentrate practically did not lead to a change in the fatty acid composition of major fatty acids. The resulting amaranth protein concentrate can be considered a source of ω-6 linoleic acid.

Table 5 presents the results of determining the main polyphenolic compounds in the composition of the original amaranth flour and the obtained protein concentrate.

The main hydroxycinnamic acids in the amaranth flour sample were caffeic, ferulic, and p-coumaric. In the amaranth protein concentrate, only ferulic acid was found, and it was concentrated during the process of obtaining the concentrate. The main flavonoids in amaranth flour were rutin and nicotiflorin (kaempferol-3-rutinoside). No flavonoids were found in amaranth protein concentrate.

Table 6 shows the relative content of saponins obtained by comparing the signal intensities of individual saponins in the composition of the original amaranth flour and the obtained amaranth protein concentrate prepared under identical conditions.

Saponins were not completely removed during the process of amaranth protein concentrate preparation.

### 3.2. Results of the In Vivo Study

The next step in assessing the biological value of amaranth protein was a quantitative evaluation of its true digestibility in experiments on laboratory animals. The protein content determined by the Kjeldahl method in the control diet was 21.7%, while it was 21.8% in the experimental diet with amaranth protein concentrate.

The rats of all groups grew normally throughout the experiment. The general condition of all animals in terms of appearance, fur quality, and behavior during daily examinations was satisfactory.

The average food intake for animals in the control group (1) for the entire period was 17.3 ± 0.3 g, while it was 16.5 ± 0.5 g for animals in the experimental group (2).

**Figure 2:**
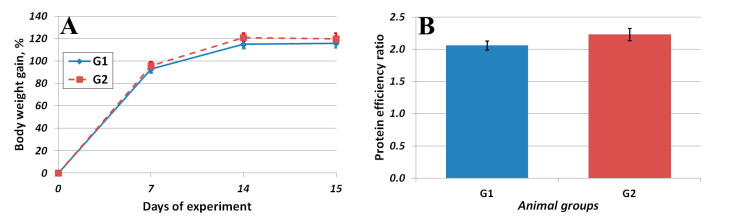
Body weight gain, % (**A**) of animals; average protein efficiency ratio values (**B**).

Figure 2A shows the dynamics of the average body weight of rats that received casein and amaranth protein concentrate as part of isocaloric semisynthetic diets for 15 days. The efficiency of utilization of amino acids in the composition of amaranth protein concentrate was characterized by an increase in body weight of the laboratory animals in grams per 1 g of protein consumed—the protein efficiency ratio (PER, Figure 2B). At the end of the experiment, the average weights of the animals in the first and second groups were 253 ± 7 g and 250 ± 10 g, respectively; the difference between groups was not significant.

No significant differences were found between the protein efficiency ratios of casein and amaranth protein concentrate.

A comparative assessment of the effectiveness and true digestibility of casein and amaranth protein concentrate is presented in Table 7.

The results of determining the true digestibility indicate that the average amount of protein consumed by rats per day in the “metabolic” period did not differ significantly between the animals of the two groups. The weight of feces excreted by rats that consumed amaranth protein concentrate was more than twice that of animals consuming casein. The values of true digestibility for casein and amaranth protein concentrate were 99.3 ± 0.2 and 97.6 ± 0.3, respectively (a statistically significant difference). The digestibility coefficient of amaranth protein concentrate, defined as the ratio of its true digestibility to the true digestibility of casein, was equal to 0.98, which is in accordance with the balance studies; hence, the value of 0.98 (98%) should be considered an estimate of the lower limit of the biological value.

## 4. Discussion

Amaranth protein isolates and concentrates are obtained from whole amaranth grain using a complex of physicochemical methods: grain grinding into flour, sifting, extraction at high pH values, defatting, centrifugation, isoelectric precipitation, and protein product drying [26]. In the vast majority of studies, defatting of the flour is carried out at the first stage of obtaining protein concentrates from plant raw materials. N-Hexane or petroleum ether are the most commonly used solvents. We eliminated the defatting stage in order to preserve the biologically active squalene and to avoid the use of toxic solvents. The squalene content in the obtained concentrate was 21.4 ± 3.0 mg/100 g.

According to [27], the isoelectric point value for the amaranth protein isolate, which ensures its most complete precipitation, is pH 4.5. The solubility of amaranth protein increases during extraction under alkaline conditions. Amaranth proteins were extracted in the pH range from 8 to 11 and precipitated at pH 4.5–5.0. With an increase in the pH of the extraction, the protein content in the obtained products increased and varied in the range of 54–80% [28]. The use of biotechnological methods for the destruction of polysaccharides in the composition of the grain during the purification of proteins from starch-containing components and membrane technologies for desalting the reaction mixture makes it possible to exclude the use of aggressive reagents [29]. In our work, amaranth was pretreated with the enzymes Celloviridin G20X and Prozyme 4G in selected optimal dosages and ratios for the destruction of ballast polysaccharides, followed by extraction of the forming mixture, precipitation of the protein by ultracentrifugation, extraction of non-precipitated protein by microfiltration of the supernatant, and a combination of protein fractions and freeze-drying. The technology we used made it possible to obtain a protein concentrate with a relatively high protein content (72.2 ± 0.6%), low Na content (255.6 ± 5.1 mg/100 g), and high content of the essential trace elements Cu and Se, as well as containing squalene. The enzymatic treatment allowed us to avoid the repeated step of protein solubilization in dilute alkali, which is usually used to obtain concentrates with a high protein content. Membrane filtration allowed us to remove the added sodium and concentrate the microelements. Eliminating the defatting step made it possible to preserve squalene and to avoid using toxic solvents.

Almost all scientific publications emphasize the high biological value of amaranth grain protein, which is determined by its balanced amino acid composition. Nevertheless, the data presented in the works of various research centers indicate that the protein amino acid score for different amaranth breeds varies in a fairly wide range [6]. There are amaranth species and breeds with whole protein that meet human needs for essential amino acids, which are therefore promising for use in food, even as the sole source of protein. At the same time, breeds with limited protein, according to the content of such amino acids as valine, leucine, isoleucine, etc., can be used in compositions according to the principle of mutual enrichment (i.e., based on the complementarity of the amino acid composition). Obviously, for a correct assessment of the prospects of using amaranth protein in human nutrition, one should take into account its digestibility (determined in vivo) and the biological value of the protein, which is currently recommended to be calculated by the PDCAAS. For example, in [8], the amino acid composition, net protein utilization, true digestibility, biological value, and PDCAAS for proteins of the whole grain flour of four amaranth breeds were determined. Valine was the limiting amino acid in all four studied amaranth breeds, and PDCAAS values varied in the range of 23.69–36.19%.

In our study, the amino acid score of the obtained amaranth protein concentrate was evaluated. It was shown that the concentrate was limited primarily by valine, which had an amino acid score of 74%. The evaluation of the true digestibility of amaranth protein concentrate was conducted in vivo; the value was 97.6 ± 0.3%, which was significantly lower than that of casein (99.3 ± 0.2%). The protein efficiency ratio value is directly proportional to the body weight gain of the rats and inversely proportional to the amount of protein eaten by the rats. PER was determined by the body weight gain of each animal that consumed our protein, and the average value was calculated. The ratio of amaranth concentrate PER/casein PER was 0.11 ± 0.1. This fact indicates that the efficiency of the amaranth protein concentrate utilized by the animals was slightly more than 100% of the efficiency of casein utilization. This is another factor that determines the high biological value of the developed amaranth protein concentrate. The PDCAAS value of the concentrate was 72.2%. The PDCAAS values calculated for the amaranth protein concentrate were low compared to the casein diet (100%), but similar to those of soybeans (78%) and significantly higher than the values of cereals (58.5%) [8].

A summary of the data obtained indicates a relatively high biological value of amaranth protein concentrate. Thus, the obtained concentrate can be used as an independent protein product. Furthermore, it is possible to propose a scheme for its mutual enrichment in valine, for example, with chicken egg protein.

The content of mineral substances in amaranth grain varies depending on the species, type of soil, mineral composition of the soil, and type of fertilizer used [30]. The whole amaranth grain is known to be a rich source of bioavailable manganese, selenium, and iron [17,31], which was confirmed by our own data (Table 3). We did not find information on the content of these elements in amaranth protein isolates in the available scientific publications. During amaranth protein concentrate processing, the micro- and macroelement contents changed, and their redistribution occurred. At the same time, 100 g of amaranth protein concentrate contained manganese in an amount corresponding to 50% of the recommended daily intake with food, as well as iron in an amount corresponding to slightly more than 90% of the recommended daily intake with food for men and more than 50% for women. The obtained concentrate was a rich source of selenium and copper.

Amaranth grain contains a unique set of phytonutrients—compounds that protect plants from aggressive environmental influences, including fungal, bacterial, and viral infections. The phytonutrients of grains include saponins, phenolic compounds (including flavonoids), phytosterols, and other compounds. For example, the total amount of phenolic acids in *A. caudatus* grains was found to be 16.8–59.7 mg/100 g [31]. The leafy parts of the plants contained many times higher amounts of flavonoids than the grain. There were also remarkable variations between species and even between varieties belonging to the same species. In our work, we found only ferulic acid in the amaranth protein concentrate, but it was in an amount even greater than in the original flour. Castel et al. also evaluated the total phenol content of *A. mantegazzianus* protein concentrate and found that acid pretreatment and ultrafiltration resulted in the removal of phenolic compounds [32]. The work of [10] also shows that some phenolic compounds that were initially present in raw materials in a soluble form, as well as phenolic compounds bound with protein, were lost during amaranth protein concentrate production. The authors explained this result by the disrupted interaction between polyphenols and proteins at pH = 9.

Herbaceous and food plants of the *Amaranthaceae* genus can be considered promising and highly available sources of biologically active triterpene saponins [33]. Saponins, along with other secondary plant metabolites, have been described as the main biologically active components of amaranth. To date, only triterpenoid saponins have been described in amaranth. Glucose and glucuronic acid are the most common monomers in the sapogenins of plants of the *Amaranthaceae* genus [34]. Mixtures or individual saponins exhibit various proven biological activities in vitro and in vivo, including immunomodulatory, hepatoprotective, antidiabetic, hypolipidemic, antiosteoporotic, antiviral, antifungal, and anthelmintic [25]. Saponins have a bitter taste, and depending on their structure, some can have a negative effect on animals and humans [33]. For example, they can cause emulsification of erythrocyte membrane lipids, causing Na+/K+ imbalance. The structure of the saponin and the number and structure of its active groups affect its membrane permeability and hemolytic ability [35]. Their quantities were significantly reduced during preparation, and they were preliminarily eliminated during industrial processing. We identified 15 saponins in total in the original amaranth flour, mainly of the bidesmoside type, whose sapogenins are related derivatives of oleanolic acid. They were not removed completely during the process of obtaining amaranth protein concentrate.

## 5. Conclusions

Scaling of the proposed and developed method of isolating protein from amaranth flour, which increased its content 2.5 times, will make it possible to obtain a concentrate of this protein for use as an ingredient in functional food products with a high biological value. These products can be recommended in the preventive nutrition of people with disorders of carbohydrate and/or lipid metabolism (cardiovascular disease, obesity, hypertension, metabolic syndrome, and type 2 diabetes) and people suffering from celiac disease, as well as in healthy nutrition, to improve the performance and health of various categories of people under adverse stress effects of various origins.

## Figures and Tables

**Table 1:** Chemical composition of amaranth flour and amaranth protein concentrate.

Sample	Content, %				
	Fat	Protein	Ash	Moisture	Carbohydrates
Flour (30% protein)	5.6 ± 0.1	28.7 ± 2.8	6.12 ± 0.06	4.1 ± 0.1	50.4 ± 2.6 including dietary fiber (unsoluble/soluble) 15.8 ± 1.5/10.2 ± 1.0
Concentrate	15.0 ± 1.0	72.2 ± 0.6	1.8 ± 0.2	3.4 ± 0.1	7.6 ± 0.8

**Table 2:** Amino acid score.

Amino Acid	Amino Acid Scale of Ideal Protein (FAO/WHO), g/100 g	Amino Acid Content in Amaranth Protein Concentrate, g/100 g	Amino Acid Score, %
Threonine	2.3	4.5	196
Cysteine + Methionine	2.2	4.0	182
Valine	3.9	2.9	74
Isoleucine	3.0	2.7	90
Leucine	5.9	6.8	115
Tyrosine + Phenylalanine	3.8	7.7	203
Lysine	4.5	6.9	153
Tryptophan	0.6	2.1	350

**Table 3:** The content of micro- and macroelements in the composition of amaranth flour and amaranth protein concentrate.

Element	Daily Norm for Men/Women, mg/day	Flour Content, mg/100 g	Percentage of Daily Requirement, %	Protein Content in Amaranth Protein Concentrate, mg/100 g	Percentage of Daily Requirement, %
Ca	1000	269.5 ± 19.7	27	75.7 ± 4.2	8
Cu	1	1.49 ± 0.02	149	5.11 ± 0.03	511
Fe	10/18	21.9 ± 0.3	219/122	9.13 ± 0.15	91/51
K	3500	2633.6 ± 17.2	75	586.9 ± 8.3	17
Mg	420	820.8 ± 26.9	195	68.5 ± 0.7	16
Mn	2	6.4 ± 0.1	320	1.00 ± 0.01	50
Na	1300	6.7 ± 0.2	0.5	255.6 ± 5.1	20
P	700	1592.1 ± 51.3	227	566.0 ± 10.7	81
Zn	12	4.71 ± 0.04	39	2.95 ± 0.01	25
**Element**	**Daily Norm for Men/Women μg/day**	**Flour Content, μg/100 g**	**Percentage of Daily Requirement, %**	**Protein Content in Amaranth Protein Concentrate, μg/100 g**	**Percentage of Daily Requirement, %**
Co	10	13.0 ± 0.2	130	23.3 ± 0.1	233
Cr	40	49.1 ± 1.5	123	183.6 ± 11.7	459
Se	70/55	76.2 ± 1.6	109/139	160.2 ± 1.3	229/291

**Table 4:** Fatty acid profile in amaranth protein concentrate and flour.

Fatty Acid	Fatty Acid Index	Fatty Acid Content, % of Total Fatty Acids	
		Original Flour	Amaranth Protein Concentrate
Lauric	12:0	0.05	0.02
Myristic	14:0	0.26	0.21
Pentadecanoic	15:0	0.11	0.09
Pentadecene	15:1	0.00	0.06
Palmitic	16:0	19.76	20.09
Hexadecene	16:1	0.06	0.05
Palmitoleic	16:1 9-cis	0.10	0.10
Margarine	17:0	0.13	0.12
Heptadecene	17:1	0.04	0.00
Stearic	18:0	3.50	3.43
Oleic	18:1 9-cis	23.41	22.95
Vaccenic	18:1 11-trans	1.12	1.05
Iso-octadecadienoic	18:2 9-trans, 12-trans	0.16	0.19
Cis, translinolic	18:2 9-cis, 12-trans	0.08	0.10
Linoleic	18:2	48.39	48.89
α-Linolenic	18:3 ω-3	0.95	0.99
Arachidic	20:0	0.76	0.70
Gondoinic (total isomers)	20:1	0.25	0.27
Eicosadienoic	20:2	0.04	0.04
Behenic	22:0	0.35	0.34
Lignoceric	24:0	0.30	0.27
Total fatty acids		99.86	99.96

**Table 5:** Content of polyphenolic compounds, µg/100 g.

Content, µg/100 g	Sample	
	Amaranth Flour	Amaranth Protein Concentrate
Caffeic acid	610 ± 30	n/d
Ferulic acid	900 ± 50	3090 ± 100
p-Coumaric acid	130 ± 10	n/d
Total hydroxycinnamic acid derivatives	7570 ± 280	3090 ± 100
Rutin	1370 ± 80	n/d
Nicotiflorin (kaempferol-3-rutinoside)	800 ± 30	n/d
Total flavonoids	2170 ± 120	-

**Table 6:** Relative saponin content in amaranth samples according to signal intensity: >5 × 10^5^–10^7^—major component, 10^4^–5 × 10^5^—average content, 10^3^—minor component.

No	Saponin	[M − H]-	Amaranth Flour	Amaranth Protein Concentrate
1	28-O-β-D-glucopyranosyl ester of 3-O-α-L-rhamnopyranosyl(1→3)-β-D-glucuronopyranosyl-2β,3β-dihydroxyolean-12-en-28-oic acid	955.54		n/d
2	28-O-β-D-glucopyranosyl ester of 3-O-β-D-glucuronopyranosyl-2β,3β,6α-trihydroxyolean-12-en-23-al-28-oic acid	823.48		
3	28-O-β-D-glucopyranosyl ester of 3-O-α-L-rhamnopyranosyl(1→3)-β-D-glucuronopyranosyl-2β,3β-dihydroxy-30-norolean-12,20(29)-diene-23-al-28-oic acid	953.47		
4	Nonidentified saponin *	962.46		
5	28-O-β-D-glucopyranosyl ester of 3-O-β-D-glucopyranosyl(1→3)-β-D-glucuronopyranosyl-2β,3β,6α-trihydroxyolean-4-desmethyl-12-en-23-al-28-oic acid	971.55		
6	28-O-β-D-glucopyranosyl(1→3)-β-D-glucuronopyranosyl ester of 2β,3β,6α-trihydroxyolean-12,20(29)-diene-28-oic acid	825.54		n/d
7	28-O-β-D-glucopyranosyl ester of 3-O-β-D-glucuronopyranosyl-2β,3β,6α-trihydroxyolean-12,20(29)-dien-23-al-28-oic acid	839.44		
8	28-O-β-D-glucopyranosyl ester of 3-O-α-L-rhamnopyranosyl(1→3)-β-D-glucuronopyranosyl-2β,3β-dihydroxy-30-norolean-12,20(29)-diene-28-oic acid	939.45		
9	28-O-β-D-glucopyranosyl ester of 3-O-α-L-rhamnopyranosyl(1→3)-β-D-glucuronopyranosyl-2β,3β-dihydroxyolean-12,20(29)-dien-23-al-28-oic acid	969.58		
10	28-O-β-D-glucopyranosyl ester 3-O-α-L-rhamnopyranosyl(1→3)-β-D-glucuronopyranosyl-2β,3β-dihydroxyolean-12,20(29)-diene-28-oic acid	955.53		
11	28-O-β-D-glucopyranosyl ester of 3-O-α-L-rhamnopyranosyl(1→3)-β-D-glucuronopyranosyl-3β-hydroxyolean-12-en-28-oic acid	939.56		
12	3-O-α-L-rhamnopyranosyl(1→3)-β-D-glucuronopyranosyl-2β,3β-dihydroxyolean-12,20(29)-diene-28-oic acid	793.35		
13	3-O-β-D-glucuronopyranosyl-2β,3β,6α-trihydroxyolean-12-en-23-al-28-oic acid	661.38		
14	3-O-β-D-glucuronopyranosyl-2β,3β,6α-trihydroxyolean-23-al-28-oic acid	677.28		
15	28-O-β-D-glucuronopyranosyl ester of 2β,3β,6α-trihydroxyolean-12,20(29)-diene-28-oic acid	663.56	n/d	
16	3-O-α-L-rhamnopyranosyl(1→3)-β-D-glucuronopyranosyl-2β,3β-dihydroxyolean-12-en-23-al-28-oic acid	807.43		

* Found in a mixture of grains of several amaranth species [25].

**Table 7:** Results of the metabolic experiment.

Parameter	Animal Groups	
	Control	Amaranth
Feces, wet weight, mg	1.0 ± 0.1	2.3 ± 0.2 *
Protein in feces, %	4.7 ± 0.2	5.4 ± 0.2 *
Nitrogen in feces, mg per wet weight	46.7 ± 6.7	121.8 ± 12.7 *
Food intake, g	20.7 ± 0.9	20.7 ± 1.2
Protein intake, g	4.5 ± 0.2	4.5 ± 0.3
True digestibility, %	99.3 ± 0.2	97.6 ± 0.3 *
PDCAAS, %	100.0	72.2

* differences are significant compared to the control group (*p* < 0.05). PDCAAS, protein digestibility-corrected amino acid score.

## Data Availability

The data presented in this study are available on request from the corresponding author.
